# Environmental impact of exhaust emissions by Arctic shipping

**DOI:** 10.1007/s13280-017-0956-0

**Published:** 2017-10-24

**Authors:** Christian Schröder, Nils Reimer, Peter Jochmann

**Affiliations:** Hamburgische Schiffbau-Versuchsanstalt GmbH, The Hamburg Ship Model Basin, Bramfelder Straße 164, 22305 Hamburg, Germany

**Keywords:** Air pollution, Arctic shipping, Climate change, Environment, NSR

## Abstract

Since 2005, a dramatic decline of the Arctic sea-ice extent is observed which results in an increase of shipping activities. Even though this provides commercial and social development opportunities, the resulting environmental impacts need to be investigated and monitored. In order to understand the impact of shipping in arctic areas, the method described in this paper determines the travel time, fuel consumption and resulting exhaust emissions of ships navigating in arctic waters. The investigated case studies are considering ship particulars as well as environmental conditions with special focus on ice scenarios. Travel time, fuel consumption and exhaust gas emission were investigated for three different vessels, using different passages of the Northern Sea Route (NSR) in different seasons of years 1960, 2000 and 2040. The presented results show the sensitivity of vessel performance and amount of exhaust emissions to optimize arctic traffic with respect to efficiency, safety and environmental impact.

## Introduction

Since 2005, an observable decrease of the Arctic sea ice extent, peaking in a record minimum in 2011–2012 during Arctic summer period. These circumstances generated a high interest in establishing new trade routes. Ensuring exploration, access and extraction of resources in this environment is of great value concerning the prospective trend of offshore engineering and economy.

Even though increasing Arctic shipping may provide commercial and social development opportunities, the resulting environmental impacts need to be investigated in detail. Several studies have assessed the potential impacts of international shipping on climate and air pollution (Derwent et al. 2005; Eyring et al. 2005) and have demonstrated that ships contribute significantly to global climate change and health impacts through emission of many pollutants such as carbon dioxide (CO_2_), methane (CH_4_), nitrogen oxides (NO_*x*_), sulphur oxides (SO_*x*_), carbon monoxide (CO) and various species of particulate matter (PM) including organic carbon (OC) and black carbon (BC). Although at present the shipping in the Arctic Ocean provides a relatively small proportion to the global shipping emissions, regional effects from substances such as BC and ozone (O_3_) become important to be quantified and understood.

The environmental conditions ships meet during operation affect the vessel’s total resistance and consequently the fuel consumption rate and the corresponding exhaust emissions. Vessels operating in head seas, strong wind and ice conditions experience more resistance and consequently burn more fuel to maintain the same speed as vessels operating in calm seas and sheltered waters. The vessel’s operating scenario and profile such as speed, routing, manoeuvers, laytime and the use of auxiliary systems affects the overall fuel consumption as well.

In this article, the performance dependency is analysed for four different possible routes of the Northern Sea Route (NSR): (1) near shore route passing mainly south of the major Russian islands, (2) one intermediate route passing in between the larger Russian islands, (3) northern route and(4) one transpolar passage (TPP) crossing the North Pole. We consider several vessels of various ice classes for three periods in the time frame from 1960 to 2040. The environmental conditions used for the study are based on the CMIP5 climate model. The results of the different case calculations are discussed and a possible trend of emissions due to shipping along the NSR from the past to the future is given.

## Materials and methods

**Fig. 1:**
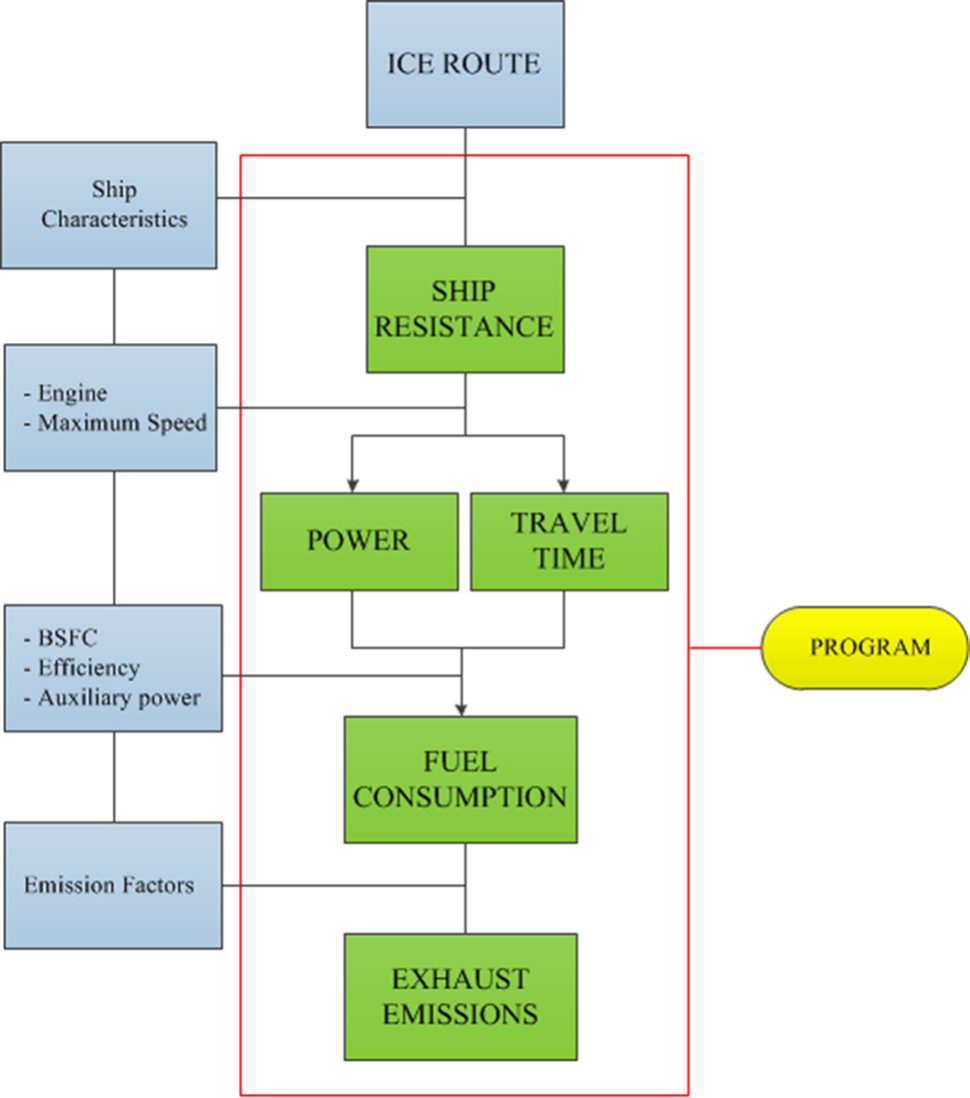
Flow chart of the calculation process (Duong 2013)

The routing programme ICEROUTE (HSVA 1999, 2014a, b) is based on semi-empirical analytical formulas for predicting ship resistance in different environmental conditions including ice coverage. Data on a specific propulsion arrangement (i.e. engine, shaft and propeller) are used to calculate the required power and thereby obtain the maximum attainable speed. The routes are divided into a number of legs according to the spatial resolution with regard to variations in environmental conditions. Different ice conditions as brash ice, broken (floe) ice and ridges (no data available in the used climate model) are related to an equivalent level ice thickness as an input parameter for the calculations. In a second step, the travelling time for the entire route can be determined by summation of travel time of each leg. In a third step, the fuel consumption can be determined based on specific engine data. Finally, the exhaust emissions are calculated based on empiric emission factors for the consumed fuel. The calculation steps and required input data are shown in the flow chart presented in Fig. 1.

### Fuel consumption of ships in ice conditions

Marine vessels are typically designed to specific operation profiles, namely a service speed that is to be achieved in environmental conditions (e.g. wind, sea state, ice) over a certain distance. The selection of main engine type and size is optimized to meet the demand of this operational profile. Newly built vessels usually have a better fuel efficiency compared to older vessels. Main reasons for this are hull deterioration and fast developments in the optimization of engine technology improvements.

Besides their impact on ship resistance the environmental conditions will also affect the efficiency of the main engine—propeller combination. A ship that operates at moderate speed in strong head sea, wind or in ice might show a similar fuel burning rate like a ship operating at relatively high speed in calm water though sailing significantly less distance within the same time.

The fuel consumption of vessels navigating in ice-covered waters depends, in addition to the sea ice conditions, on the vessel type and its ice-breaking capability, navigation instructions by authorities and ship owner or management as well as the use of auxiliary systems.

To investigate the fuel consumption of a vessel, three different methods can be used: numerical calculation and prediction, actual trial measurements of fuel consumption rates or back-calculation from fuel expenses per trip.

The brake-specific fuel oil consumption (BSFC) is a measure of fuel efficiency for reciprocating engines given by the engine manufacturer. BSFC is a possibility to directly compare the fuel efficiency of different reciprocating engines. In the following equation, the definition of the BSCF is given.

1 $$ {\text{BSFC = }}\frac{r}{P}. $$

The fuel consumption rate r is measured in grams per hour (g/h), while p is the power produced in kilowatts (kW).

There are many other factors that influence the mass fuel consumption of a vessel. An average data set analysed from over 600 measured cases of a container ship at sea is used to derive an average line to correct the BSFC and relates to the actual power of the vessel.

Practically, we use this correlation between the BSFC and the actual power when we calculate the fuel consumption of all ships. The correlation can be expressed in form of the following equation (Borkowski et al. 2011):

2 $$ \Delta {\text{BSFC}} = 6610.6x^{6} - 20524x^{5} + 23791x^{4} - 11985x^{3} + 1803.2x^{2} + 340.12x - 36.733. $$

Here, *x* = P/P_MCR_ denotes the ratio of actual power and the maximum continuous rate power. The variation $\Delta {\text{BSFC }}$ corrects the BSFC to the part load behaviour and is added to the standard value of the engine manufacturers.

### Exhaust emissions from ships

Since the 1990s, International Maritime Organization (IMO), European Union (EU), and the United States Environmental Protection Agency (EPA) came up with the Tier I exhaust gas emission norm for the existing engine in order to reduce nitrogen oxides and sulphur oxides [IMO Resolution MPEC.177 (58)]. Stricter limits for these emissions have been incorporated in Tier II (in force since 2011) and Tier III (scheduled for 2021) which were later announced for new built vessels.

Diesel fuels commonly used in marine engines are a form of residual fuel, also known as dregs or heavy fuel oil (HFO). Currently, different grades of HFO are available and sulphur restrictions are only valid in emission control areas. The Arctic is currently not an emission control area but the IMO announced a global sulphur cap of 0.5 to be implemented beginning from 2020 or 2025. Heavy fuel oil—even if it contains low sulphur—is cheaper than marine distillate fuels but contain higher amount of nitrogen, sulphur and ash content. This significantly increases the amount of NO_*x*_ and SO_*x*_ in the exhaust gas emission. The diesel engine combustion process always leaves by-products of oxides of nitrogen, unburned hydrocarbons, carbon monoxides and particulate matter.

Nitrogen oxides are a group of toxic gases formed by the reaction of nitrogen and oxygen. At extremely high temperature of combustion, these two gases react to nitrogen dioxide (NO_2_) and nitrogen oxide (NO). These gases are a major source of ground level ozone and are also a significant source of acid rains and soot formation. Unburned hydrocarbons come from unburned or partially burned fuel after combustion process. These hydrocarbons are toxic in nature, having adverse effects on our health and in some cases are known to cause cancer.

Carbon monoxide is formed as an intermediate product of hydrocarbons fuel combustion due to the lack of adequate oxygen to form carbon dioxide or due to insufficiently high temperature.

### Determination of exhaust emissions

A straight forward method to calculate emissions of a ship is using emission factors as shown in Formula 3. The amount of a specific type of fuel is directly related to the emissions. The sensitive part is to derive the emission factors for the specific combination of engine and fuel. Emission factors are based on the work of Corbett et al. (2010):

3 $$ E_{ijk} = EF_{ij} *LF_{jk} *\frac{{KW_{j} }}{{\eta_{j} }}*T_{jk}, $$

where $E_{ijk }$ is the emissions of type from vessel *j* on route *k* in gram (g); $EF_{ij}$ is the emissions factor for emissions of type on vessel *j* in (g/kWh); $LF_{jk}$ is the average engine load factor for vessel *j* on route *k* and takes into account periods of manoeuvring, slow cruise and full cruise operations; $KW_{j}$ is the rated main engine power in kilowatts (kW) for vessel *j*; $\eta_{j}$ is the engine efficiency according to propulsion train loss (Required Shaft Power = $\frac{{KW_{j} }}{{\eta_{j} }}$); $T_{jk}$ is the duration of the trip for vessel *j* on route *k* in hour (h)

### Investigated scenarios

As an input for the calculation, different scenarios are simulated according to the actual traffic along the NSR. Different routes, environmental conditions and types of vessels are investigated.

**Fig. 2:**
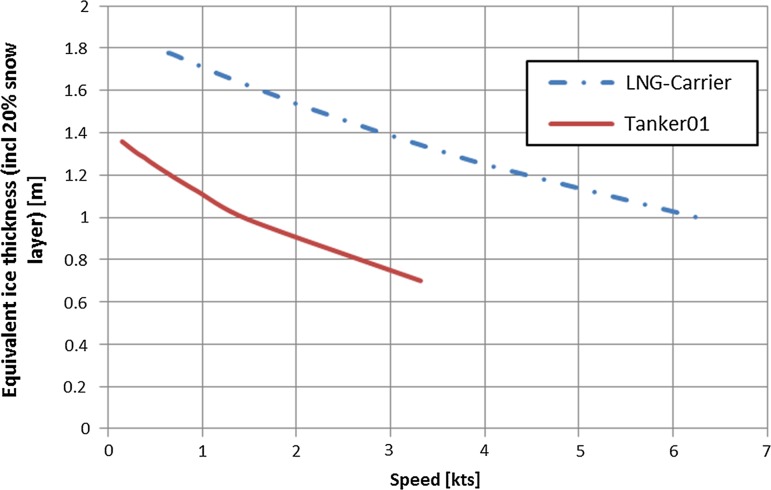
Ice thickness speed (H–V) curves at full engine load

Representative commercial ships using the NSR are currently tanker and bulker while liquid natural gas (LNG) carriers may play an important role in the future. A tanker and an LNG carrier are selected for the scenario calculations. Their main parameters are given in Table 1, while the ice thickness–speed curves (H–V curves) considering that the full engine power is used are shown in Fig. 2.

**Table 1:** Main vessel parameters

	Tanker 01	LNG Carrier
Ice class	1A Super(PC5)	Arc 7 (PC3)
Displacement (t)	102,000	115,500
LPP (m)	236	279
Breadth (m)	31.00	45.80
Propeller count, type	2, Azipod	3, Azipod
ME type	Medium speed	Medium speed, diesel-electric
Installed electric power (MW)	25	42
Required power in open water [kW]	8680.1	31075.5
Fuel grade	IFO 380	Dual fuel
BSFC (g/kWh)	190	185
Speed in open water (kts)	16	20
Safe speed in ice (kts)	8	7

Many available configurations can be considered for each ship type. With regard to the propeller type, for example, a ship can be equipped with controllable pitch propellers (CPP), fixed pitch propellers (FPP) or podded drives. In the calculations, the effects of using FPP, CPP or a podded configuration will have a strong impact on the ability of a design to maintain speed in harsh ice conditions. Vessels equipped with a FPP are commonly optimized for a single specific speed, which usually is the design condition in calm water with a small margin for heavier sea states. Thus, the FPP is over-loaded or under-loaded for certain other operation conditions. A CPP is a propeller arrangement that can adjust the blades’ pitch along their longitudinal axis. A configuration with a CPP is able to react to specific load cases and therefore avoids a reduction of the total available main engine power while a podded configuration with electric drives is able to operate at higher loads as well. Likewise more complex and thus more expensive propulsion configuration make it possible to operate efficient under very different environmental scenarios but can be less economic concerning the single propeller operating point.

Considering the engine type, the most common type would be a diesel engine fuelled with heavy fuel oil, but nowadays to fulfil the strict regulations on exhaust gases in sensitive regions (emission control areas), vessels are sometimes equipped with new kinds of engines as dual fuel configurations. Additionally, many vessels which are able to operate under heavy ice conditions are equipped with diesel-electric machinery.

**Fig. 3:**
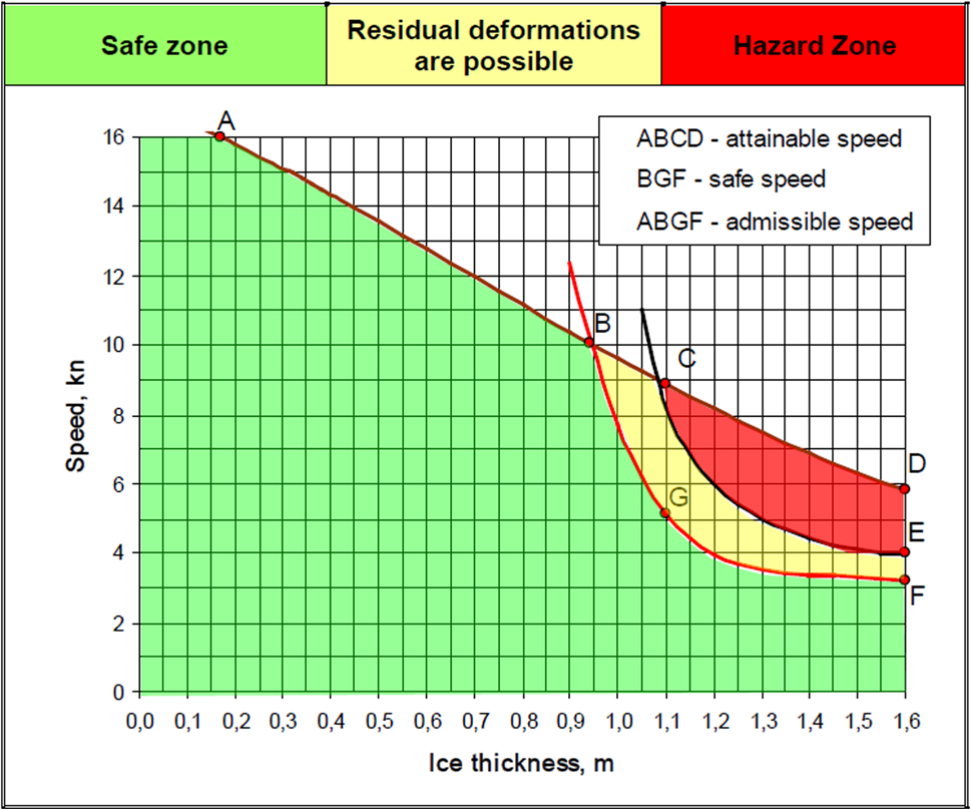
Vessel speed depending on ice thickness (CNIIMF 2007)

Figure 3 shows an example of a correlation of ship speed in different ice thicknesses. It furthermore states a curve of a safe speed for ships operating in ice-covered waters considering no ice pressure or ice ridges. As the speed of a ship in ice is limited due to higher resistance compared to open water resistance, it is necessary to further specify a safe speed in order to avoid damaging of the hull by collision with ice features sailing at low ice concentrations which allow higher speeds.

Thus, a ship travelling at a low ice concentration would potentially reach a maximum speed above the safe speed, which would result in a higher fuel consumption compared to safe speed. The regulations and permissions are described specifically for each ship in the ice certificate (Ice Passport) acknowledged by the Northern Sea Route Administration. For these scenario calculations, the safe speed curves are not available for each vessel and ice conditions. Thereby a simplified approach is used using only a single speed limitation (representing an average ice thickness) of 7–8 knots relative/to the occurrence of ice.

Additionally, an economic speed for travelling in open water is defined and used for consumption analysis in ice-free waters. Due to the safe speed and economic speed in open water, the simulations show part load conditions in most simulations.

Comparing these assumptions to a realistic crew operating the vessel, the vessel would operate with the target to minimize the travelling time but still satisfying the rules. This is not always the case as a combination of economic sailing and schedule requirements define the speed profile of the vessel. Therefore, the assumptions lead to conservative fuel consumption.

### Investigated routes

The Northern Sea Route (NSR) is a shipping lane officially defined by the Russian government from the Atlantic Ocean (Kara Gate) to the Pacific Ocean running along the Russian Arctic coast from Murmansk on the Barents Sea, along Siberia, to the Bering Strait and Far East. The entire route leads through Arctic waters. Several parts are ice free for 2 months per year. At the western end of the NSR, routes from Murmansk across the Barents Sea and Kara Sea, and up the Yenissei River to Dudinka, have been used regularly since the 1980s, but were not used by commercial transits until 2009.

Regardless of explicit routing, the NSR extends to about 3000 nautical miles. The actual length of the route in each particular case depends on ice conditions and on the choice of variants of passage resulting in individual leg-lengths.

**Fig. 4:**
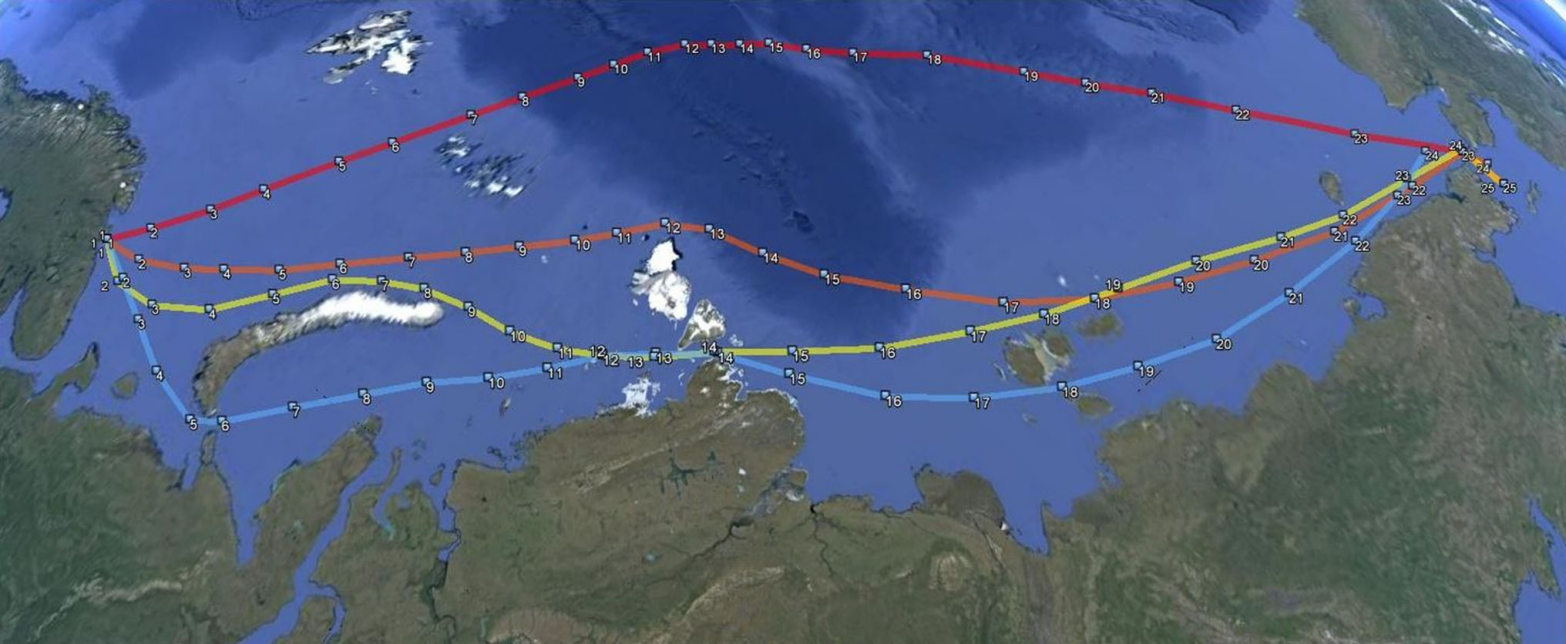
Different transit routes along the NSR: 1 near shore (blue), 2 intermediate (yellow), 3 northern (orange) and 4 transpolar (red)

The investigated route profiles can be described by the following approximate way points while several additional way points are used for the calculations (Fig. 4):

- near shore route 1 (blue, 3048 nm)—Murmansk to Bering Strait via Kara gate, south of Severnaya Zemlya and south of New Siberian Islands;
- intermediate route 2 (yellow, 2998 nm)—Murmansk to Bering Strait via north of Novaya Zemlya, south of Severnaya Zemlya and north of New Siberian Islands;
- northern route 3 (orange, 2892 nm)—Murmansk to Bering Strait via north of Novaya Zemlya, north of Severnaya Zemlya and north of New Siberian Islands;
- transpolar passage route 4 (red, 2729 nm)—Murmansk to Bering Strait via north of Novaya Zemlya, north of Severnaya Zemlya close to the geographical north pole and north of New Siberian Islands.

### Investigated ice conditions in the past, present and future

The main factor influencing navigation in Arctic waters is the presence of sea ice. The navigation season for transit passages on the NSR starts approximately at the beginning of July and lasts through to the second half of November. There are no specific dates for commencement and completion of navigation as it depends on the particular ice conditions. In 2011, the navigation season on the NSR routes for large vessels constituted 141 days in total.

**Fig. 5:**
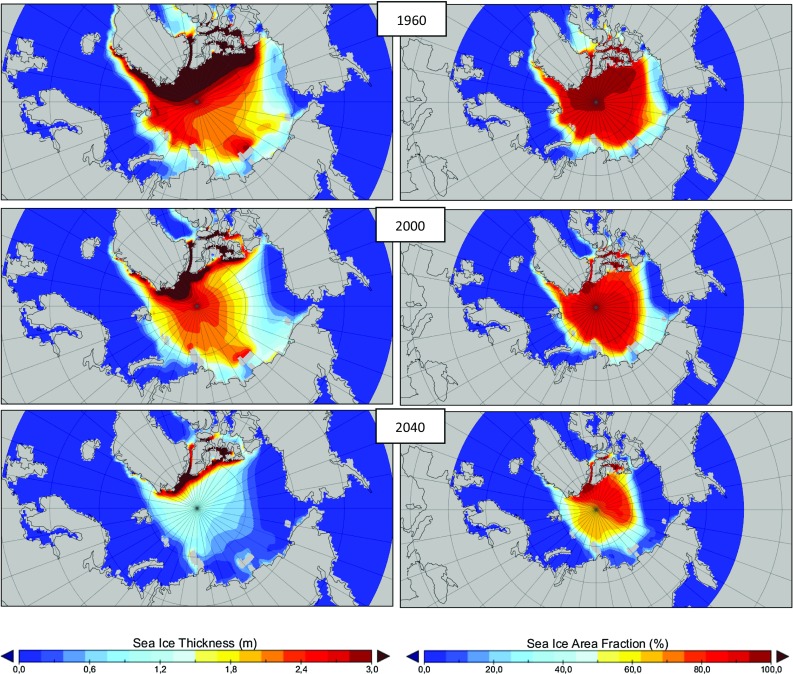
Sea ice thickness data for September 1960, 2000 and 2040 (RCP 8.5; Moss et al. 2010)

For the investigated scenarios and periods, ice data of the coupled global climate model MPI-ESM-LR (Notz et al. 2013) part of the World Climate Research Programme (WCRP) Intercomparison Phase 5 (CMIP 5) (Taylor et al. 2012) were used. The data had been obtained within the framework of the World Climate Research Program and are based on historical scenarios and different emission scenarios defined by emitted greenhouse gases in the year 2100. For the current investigation, the two scenarios RCP 4.5 and 8.5 (Moss et al. 2010) were used. The trends of ice thickness and ice concentration are shown in Fig. 5 for September 1960, 2000 and 2040.

In recent years, ice conditions offer more opportunities for operation on at the NSR. Currently, all NSR routes are located in ice regimes with first-year ice. In addition to these routes, one route is analysed which proceeds across the North Pole to investigate future ice conditions and scenarios. Under Arctic conditions, first-year ice grows approximately up to 1.6–2.0 m. In early July, at the beginning of the navigation season, ice is not pressurized. The ice is broken in floes, and vessels with suitable ice class can easily pass. In September and October, the NSR sea ways can be completely ice free. Vessels can generally achieve the same speed as in open water condition. A voyage from Cape Zhelaniya on Novaya Zemlya to the Bering Strait can be performed at a speed of 14 knots within 8 days taking into concern an almost ice-free voyage.

## Results

In total, three different vessel types, tankers, bulkers and LNG carriers, with different ice navigation capacities, ice-going and ice-breaking, were investigated. All four routes presented were examined for ice conditions occurring in April, July, September and November in the years 1960, 2000 and 2040, respectively. Results are compared and presented only for an ice-going crude oil tanker (ice class PC5) and an ice-breaking LNG carrier (ice class PC3).

In Table 2, travel time and fuel consumption are presented for both vessels for a total voyage in September (green coloured lines) on Route No. 1 for the years 1960, 2000, 2013 and 2040 while in brown colour the same data set is presented for a voyage conducted in November. For comparison purposes, the calculation results for Route No. 4 for a voyage taking place in November 2040 as well as for the commonly used Suez-Route are shown.

**Table 2:** Comparison of travel time and fuel consumption for NSR passage and Suez-Route for a complete voyage from port of Rotterdam to Port of Yokohama

Route	Tanker 01		LNG carrier	
	Time (d)	Fuel (t)	Time (d)	Fuel (t)
Route 1 September 1960^a^	26.54	983.04	25.07	1993.93
Route 1 September 2000^a^	26.44	980.94	24.71	2017.43
Route 1 September 2013^a^	25.64	777.84	24.23	1744.43
Route 1 September 2040^a^	25.14	777.44	23.55	1771.93
Route 1 November 1960^a^	163.64	13 122.34	35.79	5429.03
Route 1 November 2000^a^	52.34	3698.94	31.79	4787.63
Route 1 November 2013^a^	33.04	1980.04	25.29	3280.83
Route 1 November 2040^a^	29.54	1632.14	24.79	2878.63
Route 4 November 2040^a^	30.74	1797.78	23.75	3103.50
Suez Route	34.03	1588.02	27.23	3756.44

^a^Routes include beside the data calculated for the NSR the travel time and fuel consumption for the open water legs from Rotterdam to Murmansk and from Bering Strait to Yokohama. Open water speed is 16 knots for the Tanker and 20 knots for the LNG Carrier. Please note that on the NSR passage in case of the presence of ice due to safety reasons the speed is limited to 8 knots for both vessels

For the September voyages, the data show almost no difference between 1960 and 2000; the same is valid for 2013 and 2040. A significant difference between these two groups can be seen in the fuel consumption while only minor variations occur for the travelling time. This finding can be explained by the speed limit which is coming into effect in the case of presence of ice. Additionally, the comparison is based on single data points of these specific years, which may not reflect the average of the surrounding years. In contrast to remote sensing data as well as actual observations on-board of vessels during the past five years—which indicated an almost ice-free NSR in September—the sea ice model shows the presence of ice on several locations along the NSR. A comparison of the two vessels shows a minor benefit for both, travel time and fuel consumption, for the PC5 class ice-going Tanker.

**Fig. 6:**
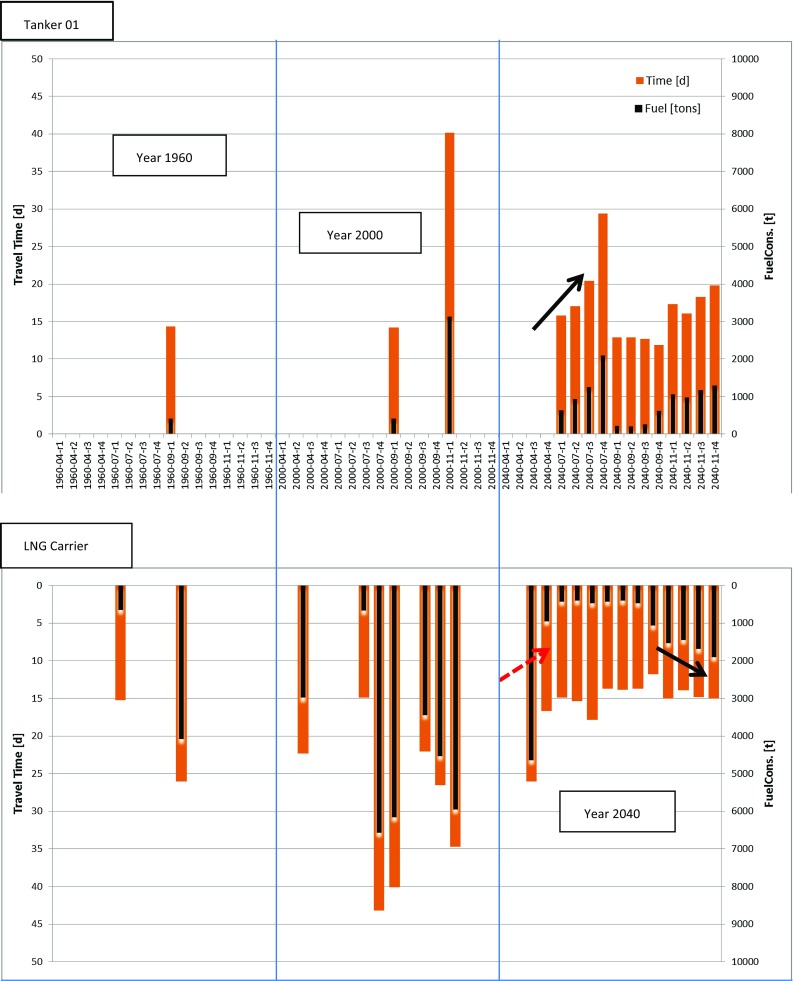
Travel time and fuel consumption for different years, months and routes (routes are defined as not completed if the travel time is exceeding 50 days; no travel time and fuel consumption is presented)

Figure 6 shows the travelling time and fuel consumption while navigating on the routes 1–4. It is obvious that Tanker 01 is not capable to finish the routes for ice conditions of the past (1960) and the present (2000) but is able to finish most of them for ice scenarios predicted for the future (2040). Routes are defined as “not completed” if the travel time exceeds 50 days. Travel times above this threshold represent ice conditions which are not applicable for the analysed vessel. The completed transits for past and present simulated ice conditions are performed mostly in September when open water is predominant and only a few ice fields with low concentration occur on the near coast routes (as for example Route 1). This allows safe and easy navigation for vessels with good open water performance, low ice class and limited installed power.

The LNG Carrier completed two transits during July and September 1960, eight transits for present ice conditions, and will be able to manage almost all ice conditions at any time in the future. This is reasonable as the vessel has a high ice-breaking capability due to optimized hull shape and 36 MW installed power.

Travel time and fuel consumption for both vessels increase with higher latitude, from coast to pole route (indicated by black arrows in Fig. 6). The increase in fuel consumption can be explained by more severe ice conditions that cannot be compensated by a shorter distance of the northern routes. In April 2040, the opposite trend is observed for the LNG Carrier (illustrated as red dotted arrow in Fig. 6). The applied climate model shows lighter ice conditions for this period along the pole route compared with the more southerly coastal routes.

The results clearly show that higher ice class than PC5 is needed for vessels navigating the NSR during other periods than August to October in the past and even at present conditions. In the future, ice class PC5 seems to be suitable to use the NSR from June to November. Vessels with ice class PC3 or higher will allow year-round transit even using the pole route according to the scenarios.

**Fig. 7:**
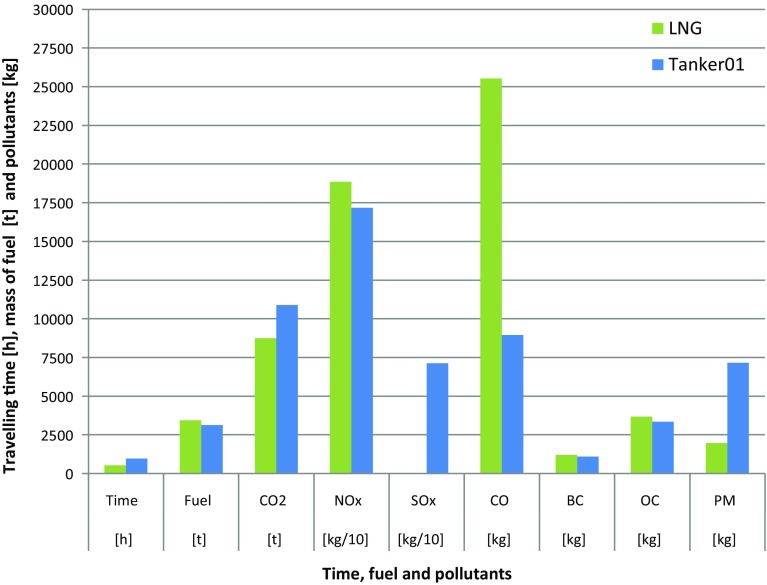
Comparison of exhaust gas emissions of Tanker01 and LNGCarrier for November 2000 using route no. 1 (2000-11-r1)

The resulting emissions will have a particular impact on the environment. We choose Route 1 in November 2000 as an example of the conditions (Fig. 7). It is important to point out that the LNG Carrier does not exhaust SO*x*, because of the dual fuel propulsion concepts, whereas ships equipped with two-stroke engines exhaust SO*x*. The ice conditions on the route chosen for this comparison are the maximum conditions for Tanker 01, which results in relatively long travel time and high fuel consumption. The LNG Carrier with significant higher ice-breaking capability has a shorter travelling time for the same route. Despite the 40% higher installed power, this vessel still consumes less fuel resulting in a lower emission. Beside carbon oxide, all other emitted components are lower for the LNG Carrier than for Tanker 01.

## Discussion

The simulations of travelling time, fuel consumption and exhaust gas emission are very sensitive to environmental parameters like ice thickness and concentration, ridge mightiness and frequency, wind speed and direction, current speed and direction and the resulting ice drift speed and direction as well as the lateral ice pressure. Furthermore, pollution calculation based on BSFC and emission factors—as well as determination of the ice resistance using semi-empirical formulas.

The global approach of the climate model gives a good overview of the general conditions in the Arctic Ocean. Predictions of the local climate, for example, in the vicinity of islands and near to the mainland shore, can be imprecise.

A tendency of decreased travel time and fuel consumption from past over present to future is observed for November passages. The outcomes emphasize that navigation on the NSR was impossible for an ice-going vessel with ice class PC5 in the past during the beginning of the Arctic winter but will become possible in the future. The PC3 class vessel had no problems to use NSR in the past and at present; in the future this route will be profitable when transporting goods.

The shortest of the investigated routes is the transpolar passage, TPP. Our findings show that there is no reason for a PC5 class vessel to use the TPP instead of the other route options in the future as the travel time is lower and additionally the fuel consumption higher. There is an advantage for the PC3 LNG Carrier using the TPP as travel time as well as fuel consumption are lower, navigating along the NSR.

When comparing the required travel time and fuel consumption of the Suez-Route to different scenarios on the Northern Sea Route, it becomes obvious that the main influence parameter is the occurrence of ice. All routes in September (marked green in Table 2) show shorter travel time and less fuel consumption compared to the Suez-Route (marked blue). Using the NSR in November already today saves time for a PC3 class vessel and will save even more in the future while navigating over the North Pole along the TPP. Due to the presence of sea ice, leading to a higher resistance, the vessels will consume more fuel when travelling along NSR or TPP.

## Conclusion

The investigation was performed using the programme ICEROUTE. The transit simulations show a clear trend of decreasing travelling time for all ship types using the NSR due to the decline of the Arctic sea ice extend and thickness. This finding together with fuel savings due to lower power requirement consequently results in lower exhaust gas emissions of the vessels. A comparison of the travel time and fuel consumption using the NSR compared with the Suez-Route shows clear savings regarding time and fuel already today and even more so in the future. The results are influenced by the fact that the vessel velocity is limited to a safe speed even in regions with only small ice coverage resulting in low fuel consumption and consequently small exhaust gas emissions.

By the restriction to safe speed, slow steaming scenarios lead to lower fuel consumption and exhaust gas emissions compared to conventional routes like the Suez-Route. Travelling along other routes in certain periods other than September, the fuel consumption increases rapidly for vessels with low ice class. Using the programme ICEROUTE, certain scenarios can be easily simulated and compared and the influence of the Arctic shipping on the environmental pollution can be assessed. The future forecasts denote a widening of operation windows for transits on all routes and especially in the freeze-up periods of October to November. To predict future scenarios, the sea ice forecast is the main uncertainty of the calculations. Actually, harsh and unpredictable ice conditions protect the area from pollution as a commercial service is not economically viable. In order to predict future exhausts emissions for the Arctic region, the number of ships that travel along the NSR under reasonable safe and economic conditions has to be determined. In addition to ice conditions, the number of ships will depend on the development of the region and its infrastructure as well as on freight rates and type of goods to be transported along the Northern Sea Route in both West–East and East–West directions.
